# Pharmacokinetic profile of a 24-hour controlled-release OROS^® ^formulation of hydromorphone in the presence and absence of food

**DOI:** 10.1186/1472-6904-7-2

**Published:** 2007-02-02

**Authors:** Gayatri Sathyan, Emily Xu, John Thipphawong, Suneel K Gupta

**Affiliations:** 1 ALZA Corporation, Mountain View, CA, USA

## Abstract

**Background:**

The objective of this study was to compare the pharmacokinetic profile of a novel, once-daily, controlled-release formulation of hydromorphone (OROS^® ^hydromorphone) under fasting conditions with that immediately after a high-fat breakfast in healthy volunteers. The effect of the opioid antagonist naltrexone on fasting hydromorphone pharmacokinetics also was evaluated.

**Methods:**

In an open-label, three-way, crossover study, 30 healthy volunteers were randomized to receive a single dose of 16 mg OROS^® ^hydromorphone under fasting conditions, 16 mg OROS^® ^hydromorphone under fed conditions, or 16 mg OROS^® ^hydromorphone under fasting conditions with a naltrexone 50-mg block. Plasma samples taken pre-dose and at regular intervals up to 48 hours post-dose were assayed for hydromorphone concentrations. Analysis of variance was performed on log-transformed data; for mean ratios of 0.8 to 1.2 (20%), differences were considered minimal. Bioequivalence was reached if 90% confidence intervals (CI) of treatment mean ratios were between 80% and 125%.

**Results:**

The mean geometric ratios of the fed and fasting treatment groups for maximum plasma concentration (C_max_) and area under the concentration-time curve (AUC_0-t_; AUC_0-∞_) were within 20%. Confidence intervals were within 80% to 125% for AUC_0-t _and AUC_0-∞ _but were slightly higher for C_max _(105.9% and 133.3%, respectively). With naltrexone block, the hydromorphone C_max _increased by 39% and the terminal half-life decreased by 4.5 hours. There was no significant change in T_max_, AUC_0-t _or AUC_0-∞_.

**Conclusion:**

Standard bioavailability measures show minimal effect of food on the bioavailability of hydromorphone from OROS^® ^hydromorphone. Naltrexone co-administration results in a slight increase in the rate of absorption but not the extent of absorption.

**Trial Registration:**

Clinical Trials.gov NCT00399295

## Background

Hydromorphone hydrochloride (HCl) is a semi-synthetic opioid agonist that is widely used for the treatment of severe chronic pain. Studies have shown that it is a potent analgesic with a tolerability profile similar to that of morphine and other opioid analgesic agents [1]. Dosage forms available for administration of hydromorphone include immediate-release and extended-release formulations. Immediate-release hydromorphone has a half-life of approximately 2 to 3 hours [2,3], and therefore must be administered every 4 to 6 hours to provide continuous pain control.

A novel, once-daily, controlled-release formulation of hydromorphone (Jurnista™, Janssen Pharmaceutica, N.V., Beerse, Belgium) is being evaluated in an effort to provide consistent pain relief with convenient 24-hour dosing in patients with severe chronic pain. The monophasic release of hydromorphone from the controlled-release formulation is achieved by utilization of the OROS^® ^Push-Pull™ osmotic pump delivery system (ALZA Corporation, Mountain View, CA) [4]. In initial studies, the consistent release of hydromorphone over a 24-hour period has been demonstrated in healthy volunteers [5], with steady-state plasma concentrations achieved by 48 hours (i.e., after two doses or by the third dose) and sustained throughout the 24-hour dosing interval [6]. Further evaluation also has confirmed that the pharmacokinetics of OROS^® ^hydromorphone are linear and dose-proportional for the 8, 16, 32, and 64 mg doses [7].

The objective of the present study was to characterize the pharmacokinetic profile of OROS^® ^hydromorphone following a single dose in the presence and absence of food. Food has been known to affect the absorption and disposition of several drugs, for both immediate-release and extended-release preparations. The effect of food on extended-release formulations, which typically have higher drug content compared with conventional formulation, is more worrisome. Extended-release formulations in general are designed to prolong the duration of efficacy and/or reduce drug's side effects. These advantages may be compromised in the presence of a significant food effect. However, osmotically controlled formulations are nearly insensitive to the gastrointestinal environment, including food [8,9].

Further, the effect of pretreatment with an opioid antagonist (naltrexone) on the pharmacokinetics of OROS^® ^hydromorphone was evaluated. Naltrexone acts competitively at mu, kappa, and delta receptors in the central nervous system [10] and has been shown to have an effect on the pharmacokinetics of controlled-release morphine [11].

## Methods

### Subjects

Study volunteers were required to be healthy, non-smoking adults (aged 19 to 50 years) with a body weight between 61 and 100 kg and within 10% of their recommended weight range for height and body frame, based on the 1984 Metropolitan Height and Weight Tables. At screening, subjects were required to have a negative urine test result for drugs of abuse, including cannabinoids, opiates, cocaine, ethanol, and barbiturates, and no clinically significant deviations from normal in laboratory test values.

Subjects who were intolerant of, hypersensitive to, or dependent on opioid agonists or antagonists were excluded, as were individuals who had psychological or physical dependence on opiates, developed tolerance to opiates, were known drug abusers or addicts, or had undergone drug or alcohol detoxification. Also excluded were subjects with gastrointestinal disorders; compromised cardiac, respiratory, renal or hepatic function; psychiatric abnormalities; or significant hematologic, metabolic or central nervous system disorders. Those taking any long-term medication, including prescription medications, or who had received enzyme-altering agents, recreational drugs, or an investigational agent within 30 days of beginning the study, also were excluded. Female subjects of childbearing potential were required to be following a medically recognized contraceptive program prior to and during the study. A negative pregnancy test was required each week, before the administration of study medication. All subjects were required to provide written informed consent. The study was approved by the Institutional Review Board and was carried out according to the Declaration of Helsinki and subsequent revisions.

### Study design

This was an open-label, randomized, three-way crossover study designed to compare the relative pharmacokinetic profile of OROS^® ^hydromorphone 16 mg under fasting conditions with the profile of OROS^® ^hydromorphone given immediately after a high-fat meal. The study also examined the effect of the opioid receptor antagonist naltrexone on the pharmacokinetics of OROS^® ^hydromorphone in the fasting state.

Based on the assumption that the within-subject variability is less than 20% (value guided by variability in exposure following immediate-release hydromorphone) and that there is a 5% difference between treatments, a sample size of 30 subjects was estimated to provide 80% power to demonstrate equivalence at the 0.05 level of significance.

### Interventions

Subjects were randomized to receive one of the following orally administered treatments during each of three study periods (i.e., a different treatment during each phase). Each treatment period was separated by a 7-day washout. Treatment A consisted of 16 mg OROS^® ^hydromorphone under fasting conditions, and Treatment B consisted of 16 mg OROS^® ^hydromorphone under fed conditions. Treatment C was 16 mg OROS^® ^hydromorphone under fasting conditions with a naltrexone block; naltrexone HCl 50 mg was given 12 hours before, with, and 12 hours after OROS^® ^hydromorphone. All subjects received OROS^® ^hydromorphone, whereas only those receiving Treatment C also received the naltrexone block.

The medication sequence received by each subject was determined by simple random assignment by subject number. Subjects reported to the study center each evening before an administration day and received OROS^® ^hydromorphone after an overnight fast (10 hours). All study medications were given with water (240 mL). Volunteers receiving Treatment B consumed a standardized high-fat breakfast (approximately 50% of total caloric content of the meal and 800 to 1000 calories) immediately before administration of the study medication, as recommended by FDA CDER Guidelines for Industry on Food-Effect Bioavailability and Fed Bioequivalence Studies [12].

### Plasma sampling

Venous blood samples for pharmacokinetic analysis were collected pre-dose (time 0) and at 2, 4, 6, 8, 10, 12, 16, 20, 24, 30, 36, 42, and 48 hours post-dose. Plasma hydromorphone concentrations were measured using a validated LC/MS/MS method (CEDRA Corporation, Austin, TX), covering a range of 0.05 to 10.0 ng/mL. Calibration standards prepared for each of the sample sets were used to monitor the inter-day precision of the assay. The coefficients of variation for the standards ranged from 1.9% to 11.7%. The absolute deviations ranged from 0.06% to 3.2%. Peak plasma concentration (C_max_), time to peak plasma concentration (T_max_), terminal half-life (t_1/2_), and area under the concentration-time curve for zero to time t (AUC_0-t_) and zero to infinity (AUC_0-∞_) were calculated.

### Safety assessments

Clinical laboratory evaluations were obtained at the pretreatment evaluation and before each treatment phase. Adverse events were recorded throughout the study.

### Statistical analysis

Log-transformed (ln) C_max_, AUC_0-t_, and AUC_0-∞ _data were analyzed using an appropriate analysis of variance (ANOVA) regression model. Treatments B and C were evaluated using ANOVA mean ratios and confidence intervals from log-transformed parameters, with Treatment A as the reference. The ANOVA model included the factors sequence, subject within sequence, phase, and treatment. For means within 20% (i.e., mean ratios of 0.8 to 1.2), differences were considered minimal. Bioequivalence was concluded if 90% confidence intervals (CIs) of treatment mean ratios were between 80% and 125%. T_max _was analyzed non-parametrically, without dose normalization, using the Wilcoxon signed rank sum test to compare differences between Treatments A and B, and between Treatments A and C. Data for t_1/2 _were summarized using descriptive statistics.

## Results

### Subjects

Thirty healthy volunteers (21 males, 9 females) were randomized. Baseline demographics are summarized in Table 1. One subject discontinued prematurely, without receiving any study medication, because of an adverse event that occurred after pre-opioid naltrexone administration (Treatment C). Another subject discontinued during the third study period (Treatment C), also after receiving pre-opioid naltrexone. The third subject withdrew for personal reasons.

**Table 1:** Baseline characteristics

**Characteristic**	**All Participants (*n *= 30)**
Sex, *n *(%)	
Male	21 (70)
Female	9 (30)
Race, *n *(%)	
Caucasian	27 (90)
Black	2 (7)
Hispanic	1 (3)
Age (years)	
Mean	33.1
Range	19–49
Height (cm)	
Mean	176
Range	163–191
Weight (kg)	
Mean	77.2
Range	64.5–94.5

The pharmacokinetic and statistical analyses include all available data from the 27 subjects who completed the study. Three of the 27 subjects had evaluable data only in two treatments. The exclusion of these subjects did not effect the bioequivalence conclusions, hence all data were kept in the final analysis.

### Pharmacokinetics

Pharmacokinetic parameters for OROS^® ^hydromorphone in fasted (alone and with naltrexone) and fed volunteers are shown in Table 2.

**Table 2:** Pharmacokinetic parameters for hydromorphone after administration of OROS^® ^hydromorphone to fasted and fed volunteers

**Parameter**	**Treatment Regimen A (fasting; *n *= 25)**	**Treatment Regimen B (fed; *n *= 27)**	**Treatment Regimen C (fasting with naltrexone block; *n *= 26)**
C_max _(ng/mL)			
Mean	1.107	1.352	1.635
SD	0.2058	0.3633	0.5708
T_max _(h)			
Median	16.0	12.0	12.0
Range	6.0–36.0	6.0–20.0	6.0–24.0
t_1/2 _(h)			
Mean	14.7	12.5	10.1
SD	6.07	5.22	3.95
AUC_0-t _(ng· h/mL)			
Mean	31.12	30.20	33.97
SD	7.063	8.738	9.007
AUC_0-∞_(ng·h/mL)			
Mean	38.84	36.09	37.24
SD	9.566	10.04	9.921

### Food effect (Treatment Regimen A vs. Treatment Regimen B)

**Figure 1:**
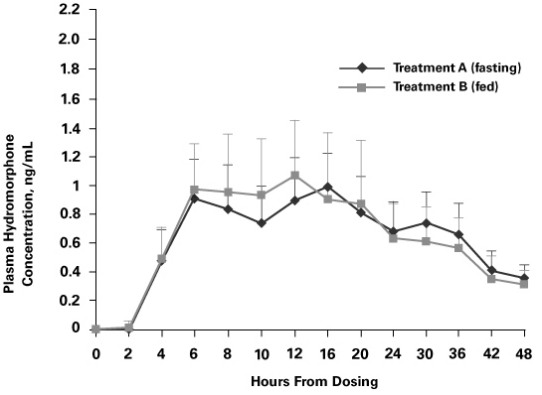
Mean plasma concentration in healthy volunteers vs. time profiles for Treatment A (OROS^® ^hydromorphone 16 mg; fasting conditions) and treatment B (OROS^® ^hydromorphone 16 mg; fed conditions).

The mean plasma hydromorphone concentrations and time profiles for OROS^® ^hydromorphone after administration to healthy volunteers under fasting (Treatment A) and fed (Treatment B) conditions are shown in Figure 1.

The mean profiles following the two treatments overlap for the first 6 hours post-dose suggesting that food has no effect on the initial rate of rise. However, mean overall C_max _increased slightly when OROS^® ^hydromorphone was administered after a high-fat meal (1.352 ng/mL vs. 1.107 ng/mL). The mean ratio of ln C_max _was 1.189, indicating that the means were within 20%. The 90% CI for the mean ratio of the product means using log-transformed data ranged from 105.9% to 133.3%.

Exposure to hydromorphone (expressed as the mean AUC_0-t_) was 31.12 ng·h/mL in the fasting state and 30.20 ng·h/mL in the fed state, whereas the mean AUC_0-∞ _was 38.84 ng·h/mL in the fasting state and 36.09 ng·h/mL in the fed state. The 90% CIs for the ratios of the product means were within 80% and 125% for both AUC_0-t _(84.9%–103.7%) and AUC_0-∞ _(81.9%; 99.4%).

Median T_max _was achieved 4 hours earlier when OROS^® ^hydromorphone was administered under fed conditions compared with fasting conditions (12 vs. 16 hours; *P *= 0.0062). The 90% CI for the difference between product medians was -6.0046 to -1.9981. Mean t_1/2 _was approximately similar under fed and fasting conditions (Table 2). The between-subject variability in hydromorphone exposure was similar for the two treatments.

### Naltrexone effect (Treatment Regimen A vs. Treatment Regimen C)

**Figure 2:**
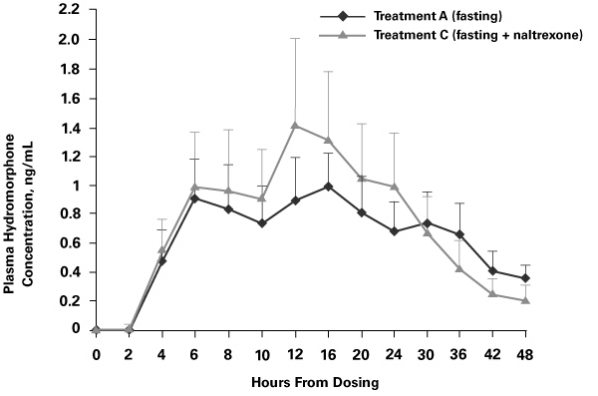
Mean plasma concentration in healthy volunteers vs. time profiles for Treatment A (OROS^® ^hydromorphone 16 mg; fasting conditions) and Treatment C (OROS^® ^hydromorphone 16 mg; fasting conditions and naltrexone block).

The mean plasma hydromorphone concentrations and time profiles for OROS^® ^hydromorphone after administration with (Treatment C) and without (Treatment A) naltrexone in fasting healthy volunteers are shown in Figure 2.

Naltrexone increased the mean C_max _from 1.107 ng/mL to 1.635 ng/mL, an increase of 39%. Moreover, the 90% CI for the ratio of the product means using log-transformed data (123.5%; 156.1%) fell outside the limits of bioequivalence (80%–125%).

Hydromorphone exposure, in terms both of AUC_0-t _and AUC_0-∞_, was similar in both treatment groups. The 90% CIs for the ratios of the product means using log-transformed data (96.7%; 118.5% and 85.0%; 103.0%, respectively) were contained entirely within the 80%–125% equivalence limits.

Median T_max _occurred 4 hours earlier when OROS^® ^hydromorphone was administered with naltrexone (12 hours) vs. without naltrexone (16 hours; *P *= 0.3493). Mean t_1/2 _was approximately 4.5 hours shorter with than without naltrexone (10.06 vs. 14.66 hours). The between-subject variability in hydromorphone exposure was similar for the two treatments.

### Safety

At least one adverse event was experienced by 24 of the 30 participants (80%). Apart from one episode of severe dizziness during Treatment C (OROS^® ^hydromorphone with naltrexone), all events were of mild or moderate intensity. The most frequently occurring adverse events were nausea and asthenia (Table 3). Abnormalities in laboratory results were minor and were not considered as adverse events by the investigator. No serious adverse events were reported during the study. Two patients discontinued the study because of adverse events after the initial naltrexone dose of Treatment C.

**Table 3:** Adverse events [*n *(%)] occurring in ≥ 10% of subjects in any treatment group

**Event**	**Treatment Regimen A (fasting; *n *= 28)**	**Treatment Regimen B (fed; *n *= 29)**	**Treatment Regimen C (fasting with naltrexone block; *n *= 27)**
Nausea	6 (21.4)	9 (31.0)	7 (25.9)
Asthenia	6 (21.4)	8 (27.6)	7 (25.9)
Dizziness	8 (28.6)	5 (17.2)	3 (11.1)
Pruritus	2 (7.1)	4 (13.8)	9 (33.3)
Headache	6 (21.4)	3 (10.3)	4 (14.8)
Vomiting	2 (7.1)	3 (10.3)	5 (18.5)

## Discussion

Food can interfere with the bioavailability of a drug via several mechanisms, including physically binding the drug, altering gastric pH, inhibiting or inducing presystemic metabolism, changing hepatic blood flow, altering drug solubility, and promoting disintegration of the drug formulation. The latter effect is particularly important for extended-release formulations because they contain larger amounts of drug than conventional formulations.

With immediate-release hydromorphone, there is a 24% increase in AUC and approximately 25% decrease in C_max _when taken with food as compared to the fasted state [13]. The effect of a high-fat meal is generally expected to be seen within the first few hours (4–6) of dosing as it interacts with the formulation in the stomach, delays gastric emptying, and may have other effects on drugs absorption and metabolism [14] or cause dose dumping due to disruption of the controlled-release mechanism of the formulation. The results of this study show that consumption of a high-fat meal immediately before dosing with OROS^® ^hydromorphone does not affect overall hydromorphone exposure, and the overall mean C_max _is within 20%. The median T_max_ was lower with food (12 hours) compared with the fasting condition (16 hours), but the mean plasma concentration profiles are generally super-imposable, especially up to 6 hours post-dose (Figure 1). These results indicate that the controlled-release properties of the formulation were maintained when taken with food and that the effect of food on hydromorphone rate and extent of exposure is minimal compared with immediate-release hydromorphone. This is in contrast to another controlled-release formulation (palladone), whose concentration increases significantly (1.5-fold) when the drug is taken with food, especially during the first 6 hours after dosing [15]. The lower median T_max _and 20% increase in C_max _with OROS^® ^hydromorphone could be attributed to delayed gastric emptying with food, resulting in the drug release in the small intestines for longer duration where the drug absorption is more efficient. Since the controlled-release nature of the formulation is not affected, the slightly lower T_max _and higher C_max _are not expected to be safety concerns for OROS^® ^hydromorphone. No differences between male and female patients were observed (data not shown); however, the small number of female participants does not allow for any definitive conclusions to be drawn.

Co-administration of OROS^® ^hydromorphone with naltrexone under fasting conditions appeared to increase the rate (but not the extent) of absorption of hydromorphone, with a 39% increase in C_max_ and a 4.5-hour reduction in t_1/2_. There was no significant change in T_max_, AUC_0-t_, or AUC_0-∞_. These results with no effect on overall drug exposure indicate that blockade of opioid effects by naltrexone is useful in comparative bioavailability studies of high-dose opioids in healthy volunteers, with the assumption that all treatments are affected similarly. The results of this study are also consistent with a previous study in which the effect of naltrexone on the pharmacokinetics of controlled-release oral morphine sulfate was assessed [11]. Naltrexone did not affect the concentration-time curve for controlled-release morphine, but the AUC_0–24_ and C_max _were increased significantly, accompanied by a significant decrease in apparent absorption half-life of morphine. The T_max _and apparent elimination half-life of morphine were not significantly affected. The dose of naltrexone used in that study was 100 mg [11]. A 50-mg dose was chosen for the present study in an attempt to minimize naltrexone-related adverse events.

Adverse events occurred in a similar proportion of patients in each treatment arm and were mild to moderate in intensity, with no serious adverse events occurring during the study. The types of adverse events reported were consistent with those expected for an opioid agonist and antagonist, primarily affecting the gastrointestinal and central nervous systems. The two patients who discontinued because of adverse events did so after receiving the first naltrexone dose (Treatment C) and before receiving OROS^® ^hydromorphone.

## Conclusion

The effect of food on the bioavailability (rate or extent of absorption) of hydromorphone from OROS^® ^hydromorphone in healthy volunteers is considered to be minimal, therefore it can be taken without regards to food. Concomitant administration of naltrexone slightly increases the rate, but not the extent, of hydromorphone absorption from OROS^® ^hydromorphone in fasting healthy volunteers.

## Competing interests

The authors declare that they have no competing interests.

Financial Competing Interests

In the past five years none of the authors of this manuscript have received reimbursements, fees, funding, or salary from an organization that may in any way gain or lose financially from publication of this manuscript, either now or in the future.

The authors of this manuscript do hold stock in ALZA Corporation but the stock price is not affected by the product alone or the publication.

There are no patents filed based on the data disclosed in this publication. The authors have not received reimbursements, fees, funding, or salary from an organization that holds or has applied for patents relating to the content of the manuscript.

The authors do not have any other financial competing interests.

The authors do not have any non-financial competing interests (political, personal, religious, academic, ideological, intellectual, commercial or any other) to declare in relation to this manuscript.

## Authors' contributions

GS reviewed the pharmacokinetic and statistical results and authored the related sections. EX performed all the pharmacokinetic and statistical analysis. SKG participated in the conception of OROS hydromorphone product and contributed to the design of the study. JT reviewed the safety results and authored the related sections. All authors read and approved the final manuscript. All authors wish to acknowledge Philip Sjostedt and PharmaGenesis, Inc., in preparation of this manuscript.

## Pre-publication history

The pre-publication history for this paper can be accessed here:


